# Applications of transposon-insertion sequencing for understanding bacterial physiology

**DOI:** 10.1099/mgen.0.001796

**Published:** 2026-09-03

**Authors:** Emily C. A. Goodall, Stineke van Houte

**Affiliations:** 1 Environment and Sustainability Institute & Centre for Ecology and Conservation, University of Exeter, Penryn, TR10 9FE, UK; 2 Institute for Molecular Bioscience, University of Queensland, Brisbane 4072, QLD, Australia

**Keywords:** functional genomics, INSeq, TraDIS, transposon mutagenesis, transposon-insertion sequencing (Tn-seq)

## Abstract

Transposon-insertion sequencing (Tn-seq) couples transposon mutagenesis with next-generation sequencing to identify the transposon insertion site for thousands of mutants in parallel. It is a powerful technology with a myriad of uses beyond the identification of essential genes required for a cell to grow and divide. Tn-seq is particularly useful as a high-throughput method to assign function to function-unknown genes, which have increased steadily with the abundance of newly sequenced bacterial genomes. Tn-seq has now been adapted for use in over 100 bacterial species. Here, we summarize the applications of Tn-seq for querying bacterial physiology and discuss some of the possible applications for the future.

**Impact Statement**

Functional annotation of bacterial genomes is lagging behind the rapid identification of genes from vast amounts of sequencing data. Understanding gene function is key to understanding bacterial physiology, whether for understanding virulence factors or exploiting bacterial proteins for biotechnological uses. Transposon-insertion sequencing (Tn-seq) is a high-throughput functional genetics screen that can address this gap. This perspective introduces the range of ways in which Tn-seq can be applied to investigate bacterial physiology and presents some challenges and future advances.

## Data Availability

No raw data was generated for this article. The national centre for biotechnology information (NCBI) literature database was searched (26/03/26) using the search terms: Title/Abstract "tradis" OR Title/Abstract "tn-seq" OR Title/Abstract "INSeq" OR Title/Abstract "transposon mutagenesis" AND MeSH Terms "bacteria", to identify bacterial species that Tn-seq has been applied to. These data are presented in Table S1.

## Introduction

 Mutating a gene and examining the effect, or phenotype, is a bedrock of molecular microbiology, from early experiments of random mutagenesis using chemical mutagens to random transposon mutagenesis and targeted mutagenesis using homologous recombination. Transposon-insertion sequencing (Tn-seq) transformed this classical approach by coupling dense transposon mutagenesis with next-generation sequencing, enabling genome-wide, high-throughput assessment of gene function at scale in diverse bacterial species. With the development of cheaper and higher quality sequencing, the depth of information available from these screens allows us to ask different questions than were possible before. In this perspective, we focus on the wide-ranging applications of Tn-seq for dissecting bacterial physiology, from mapping essential functions and conditionally essential genes to revealing genetic interactions and regulatory networks, alongside discussing current limitations and future directions.

## History of transposon mutagenesis

A transposon is a mobile genetic element that can be mobilized by its cognate transposase, an enzyme that recognizes inverted repeat sequences at the boundaries of the transposon and mediates transposition to a new genomic position [1]. Arguably the earliest work in this field was the discovery of transposons by Barbara McClintock and the identification that these mobile genetic elements can lead to gene inactivation at the site of insertion [2, 3]. This laid the foundation for the later use of transposons as a tool for insertional mutagenesis. Subsequent work in the 1970s identified that transposons often encode antibiotic resistance elements, enabling them to be used as selection markers for mutagenesis [4]. The earliest characterized transposons were Tn5 and Tn10 [5, 6], followed later by members of the *Tc1*/*mariner* superfamily [7, 8]. Subsequent research involved optimizing their use for genetic screens. This included the development of engineered transposon derivatives and protocol adaptations, such as providing the transposase externally to the transposable element to improve transposon stability or including transcriptional reporters into the transposon [9, 10]. Vectors for transposon delivery were also developed [11], paving the way for optimized, larger-scale transposon mutagenesis screens. The first library of transposon mutants was a pool of >1,000 mutants used to identify *Salmonella enterica* serovar Typhimurium mutants with attenuated virulence in mice, in a method termed signature tagged mutagenesis [12]. Tn5 mutagenesis tools were further optimized with the identification of a hyperactive transposase and an optimized 19 bp inverted repeat sequence that increased transposition efficiency [13, 14]. The ‘EZ-Tn5’ system was soon commercialized and supplied as either a ready-made transposome (transposon in complex with the transposase) or as individual components, making large-scale transposition accessible and readily available.

## Large-scale transposon-insertion sequencing

**Fig. 1.**
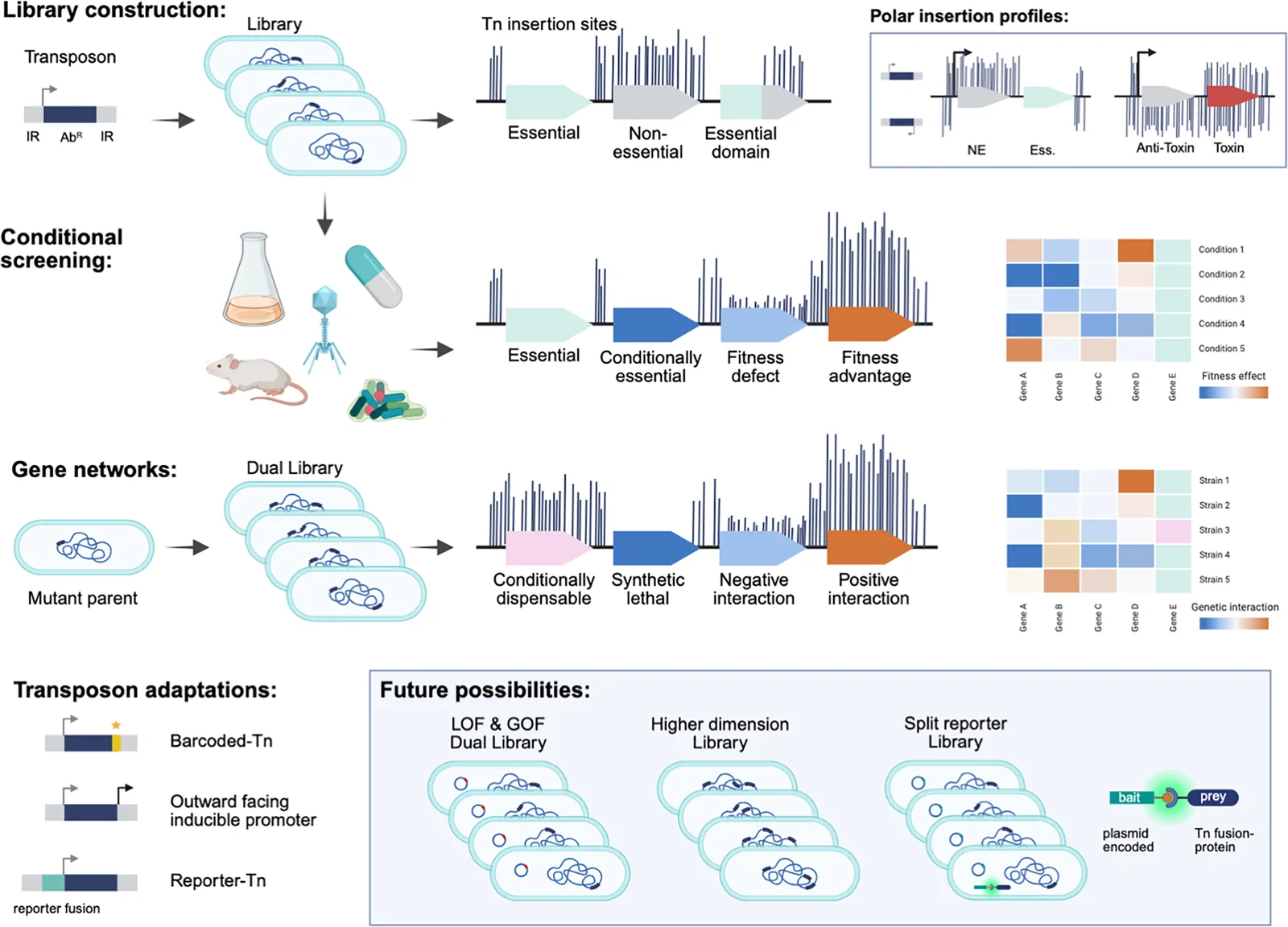
Applications of Tn-seq. Overview of the transposon (Tn) library construction process, depicting one Tn per cell integrating into the chromosome at random. Following sequencing of Tn-genomic DNA junctions, the Tn insertion site is mapped to a reference genome and these frequencies are counted, producing a graph of Tn insertion frequency across the genome for each nt position. These graphs represent a pool of mutants and a stretch of no insertions is indicative of an essential gene or domain. The library can be grown under different conditions, and changes in mutant frequency, inferred from changes in Tn–gDNA sequencing data, are indicative of positive or negative fitness effects. Abbreviations: Ab^R^, antibiotic resistance selection marker; Ess., essential; GOF, gain of function; IR, inverted repeat; LOF, loss of function; NE, non-essential.

The next significant advance came in 2009 when four methods were published that combined saturating transposon mutagenesis with the use of ‘next-generation’ short-fragment sequencing platforms to identify the transposon insertion sites in parallel at scale [15–18]. These four methods (TraDIS, Tn-seq, INSeq and HITS) and their methodological differences have been compared and extensively reviewed elsewhere [19–21]. All four methods share the overarching principle of applying random mutagenesis using a transposon to construct libraries of thousands of mutants, followed by ‘deep sequencing’ to identify the transposon insertion site. This enables the high-throughput identification of transposon insertion sites for libraries in the order of 100,000s of mutants, offering an unprecedented (approaching single bp) level of resolution into genome biology. Fundamentally, these libraries can be used to identify essential genes as disruption of any gene that is essential for viability will be lost from the population during selection or outgrowth and will be absent from the sequenced dataset (Fig. 1). In addition to genes that are essential for a bacterium to grow and divide, Tn-seq can also reveal genes that are required for plasmid maintenance and replication [22, 23]. The high resolution of Tn-seq data can offer sub-coding sequence resolution, such as identification of essential protein domains [24]. For example, FtsW is an essential peptidoglycan glycosyltransferase required for cell wall biosynthesis, but a dense transposon library in *Corynebacterium glutamicum* revealed that the *C. glutamicum* FtsW has an extended C-terminal domain, and only the glycosyltransferase domain is essential, while the C-terminal domain is dispensable [25]. The extended C-terminal domain is predicted to interact with the cell division protein FtsZ; the Tn-seq data therefore identify the essential function of the glycosyltransferase but suggest that interaction with FtsZ is dispensable in this species.

Secondly, because the transposon–gDNA junction sequencing data are single-stranded, the strand (sense or antisense) of the reference genome to which the sequencing data are mapped reveals the transposon orientation at the site of insertion. Many transposons are designed to minimize polar effects, defined as when neighbouring genes are additionally affected by transposon disruption, by maintaining transcription of any genes downstream from the transposon-disrupted gene. This feature of maintaining downstream transcription and the ability to observe transposon orientation can reveal insertional biases that are biologically meaningful [26]. For example, it is possible to disrupt upstream of an essential gene provided expression of the essential gene is maintained. Conversely, these insertional biases can also uncover antitoxin–toxin pairs if the antitoxin is encoded upstream; it is toxic to the cell to disrupt expression of the antitoxin while driving expression of the toxin. Overall, the insertional bias from polar effects can be leveraged to identify (1) co-transcribed non-essential and essential genes, (2) the position of essential promoters, (3) toxin–antitoxin pairs and (4) genes that are toxic when expressed.

## Method applications

### Phenotypic screening

Transposon libraries are especially useful for conditional screens and assigning functionality to genes of unknown function [27]. Fitness assays of pooled mutants involve exposing the pool of mutants to a selection pressure, for example antibiotic exposure, phage infection, nutrient availability or changes in temperature or pH, and using competitive outgrowth to identify more fit mutants from less fit mutants (Fig. 1). The expectation is that more fit mutants will proliferate (and therefore be more abundant), whereas less fit mutants will be outcompeted and be relatively less abundant within the total mutant pool. The fate of mutants can be tracked through sequencing the transposon-insertion sites as a proxy for mutant fitness. A benefit of this approach is that very subtle phenotypes can be identified when compared with the population of all other mutants, although this same strength can be a limitation under strong selection pressures or sustained outgrowth, when less-fit mutants are outcompeted to extinction. However, because mutants are pooled together, it is impossible to identify mutants with a function that can be complemented *in trans*. It is possible to overcome this limitation by assaying libraries in an arrayed or spatially isolated format, such as ‘droplet Tn-seq’ (discussed below [28]), although such independently assayed mutants often require the library to be of a smaller size overall due to limitations of scale.

Importantly with any competitive Tn-seq screen, fitness contributions can be quantified and ranked, allowing the strongest fitness effects to be identified and prioritized for follow-up. It is also possible to rank the contributions of essential genes: Gallagher and co-authors tracked how quickly essential gene mutants die after disruption [29]. They found that essential gene mutants were lost from the population at different rates and that mutants could largely be grouped by overall processes. For example, genes involved in nucleotide synthesis were some of the most sensitive to disruption, whereas mutants of genes involved in outer membrane protein localization took longer to be depleted from the population. Although the rate of mutant depletion is also influenced by how abundant the gene product is at the point of disruption and how quickly the product is turned over, these results nonetheless give remarkable insight into the path to cell death.

One of the limitations of transposon mutagenesis screens is that the library construction method is destructive and the library pool of mutants does not include mutants of essential genes. Other high-throughput methods such as CRISPR (clustered regularly interspaced short palindromic repeats) interference ‘CRISPRi’ might be better suited for screens where essential genes need to be included, as this gene-silencing approach is non-destructive, inducible and titratable (reviewed extensively elsewhere [30, 31]). However, modification of the transposon to include an outward-facing inducible promoter can extend standard Tn-seq assays to include gain-of-function (GOF) screening [32]. The inducible promoter drives expression of downstream genes, including essential genes, to identify fitness effects caused by changes in the expression of the downstream gene. For example, this approach can identify antibiotic targets if target overexpression confers resistance [33]. Santiago *et al*. screened a pool of transposon mutants containing an outward-facing promoter against 32 antibiotics and positively identified overexpression of *murA*, *murJ* and *fabI* as conferring resistance to fosfomycin, DMPI (a Lipid II export inhibitor) and triclosan, respectively [34]. Moreover, through a supervised machine-learning approach to cluster antibiotics based on their resistance profiles, they identified the target and mechanism of action of the antimicrobial lysocin E. Finally, libraries need not be limited to a single outward-facing promoter; Santiago *et al*. used a pool of transposons that each contained one of six outward-facing promoters of different strengths and growth-phase activity to capture variations in gene expression level [34].

Given the scale at which high-throughput functional genetics screens can be applied, one useful adaptation of the original methods is the inclusion of a barcode into the transposon, termed ‘Random Barcode’ or ‘RB-TnSeq’ [35]. After the initial characterization of the library to assign barcodes to their corresponding Tn–gDNA junction, all subsequent experiments can be analysed through PCR and sequencing of just a 20 nt barcode to track the fate of mutants.

Finally, the range and complexity of phenotypic screens have also increased over time. Tn-seq can be coupled with other methodologies, such as FACS (fluorescence-activated cell sorting) and single-cell microfluidics techniques like ‘droplet Tn-seq’. Coupling Tn-seq with FACS enables sorting of populations of bacterial cells based on morphological differences, such as cell size, or fluorescence properties, such as dye accumulation or reporter activity (discussed here: [20]). Droplet Tn-seq encapsulates single transposon mutants suspended in growth medium in oil droplets [28] and can be used to assay mutant phenotypes individually in the absence of trans-complementation. As an example, droplet Tn-seq has been used to identify the primary secretion mechanism of *Yersinia pestis* for secretion of the siderophore yersiniabactin [36]. Whether Tn-seq is further coupled with other single-cell microfluidics and imaging-based techniques remains to be seen. Secondly, Tn-seq is often limited for use in *in vivo* animal infection studies due to the combination of tight bottlenecks that impose a strong reduction in population size and/or the upper limit of the infectious dose that can be administered. Substitute *in vitro* conditions are often lacking in complexity, and bacterial factors required for *in vitro* growth might not correlate with those required *in vivo*. However, with advances in tissue engineering, more complex models such as human organoids enable Tn-seq screening under more relevant infection-like conditions [37], and the development of infection models resistant to high-dose infection enables larger screens of mutant pools *in vivo* [38]. Additionally, Tn-seq was recently adapted such that transposition of the mini-Tn5 transposon could be induced *in vivo*, enabling the generation of dense transposon libraries at a defined time point [39]. This system comprised a mini-Tn5 encoded within a Tn7 transposon, with an inducible Tn5 transposase divergently transcribed to the mini-Tn5 selection marker and outside the Tn5 inverted repeats. Following delivery of the Tn7 transposon, induction of the transposase leads to transposition of the mini-Tn5 and expansion of the Tn5 library. A major advantage of this approach is that it can bypass the selection pressure imposed by tight bottlenecks. For example, *Citrobacter rodentium* infection is normally restricted by a strong bottleneck of ~1–100 cells that initiate infection. Basta *et al*. successfully used ‘inducTn-seq’ to overcome this limitation and identified *C. rodentium* factors required for intestinal colonization in a mouse model by inducing Tn*5* mutagenesis at day 3 post-infection. They identified anaerobic and microaerobic metabolic pathways vital for infection, among others, thus demonstrating the utility of this method for *in vivo* studies.

### Gene interaction networks

A limitation of transposon mutagenesis screens is that it is challenging to uncover the phenotypic contribution of a gene whose function can be provided by a functional homologue. However, Tn-seq can also be applied for uncovering such gene relationships (Fig. 1). A gene interaction is inferred when the double mutant (i.e. where two genes are mutated) phenotype differs from the expected phenotype predicted by the behaviour of the individual mutants. For example, a synthetic lethal relationship is defined as when two genes are independently non-essential, but deletion of both is lethal to the cell. A well-known example of this in *Escherichia coli* is the two penicillin-binding proteins PBP1A and PBP1B (encoded by *mrcA* and *mrcB,* respectively). Both PBP1A and PBP1B are bifunctional peptidoglycan synthases with transglycosylase and transpeptidase activities required for peptidoglycan synthesis. Either can be deleted independently, as the function of the other can compensate, but loss of both is lethal [40]. Tn-seq can broadly be applied in two ways for identifying such gene interactions: (1) creating a library in a mutant background of interest and (2) constructing transposon libraries in multiple strains of a species to identify how the accessory gene content modifies gene fitness, in particular gene essentiality. For the former approach, construction of a transposon library in a defined mutant, for example a single gene-deletion strain, enables identification of all positive and negative interactions with the initial mutation (including the extreme negative of synthetic lethal gene pairs) on a whole-genome scale. For example, construction of a transposon library in a Δ*recG* strain validated known synthetic lethal interactions with *dam*, *uvrD* and *rnhA*, and newly identified a strong negative interaction with *rep,* overall allowing the authors to map the genetic requirements for genome maintenance [41]. With recent technological advances, it is now possible to create ‘dual libraries’ that can query gene interaction networks at scale for most gene pairs. This has been demonstrated for a dual transposon library [42] and a combined CRISPRi–Tn-seq approach [43], discussed below. In addition, two studies have also developed dual CRISPRi screens for identifying gene interactions [44, 45].

The dual-Tn approach involved construction of a library with two transposons integrated at random per cell, each with different selection markers. Briefly, a single transposon library was constructed for each selection marker independently. The transposons carried a random barcode, and the barcode for every transposon was recorded and mapped for each transposon insertion site. The DNA of one mutagenized library was then introduced via natural competency into the second library to create the dual library [42]. Previously, the biggest barrier to applying dual-Tn screens at scale was the challenge of mapping both transposon insertion sites per cell with ease. Here, a Cre-Lox system was applied to recombine the transposon barcodes into a single identifier for mapping both transposons in a single sequencing run. This method therefore enables dual random-insertion mutagenesis at an unprecedented scale, sampling almost 1 million gene pairs in a single experiment, equating to ~73% of the total possible double-mutant combinations. However, mutagenesis at this scale might be limited to species that can be transformed with relative ease. The CRISPRi–Tn-seq approach involved the construction of Tn libraries in 13 CRISPRi strains and, although smaller in scale than dual-Tn, it importantly enables the interaction mapping between essential and non-essential genes.

The second approach of constructing transposon mutant libraries in multiple strains of a species is less direct yet can still reveal gene–gene relationships. Pan-essential gene studies have been applied in several species [46–49], and all have found that there are broadly two cohorts of essential genes: those that are ‘core’ or ‘universal’ essential genes and those that are context-dependent, for example those that become non-essential when the genome contains an additional functional homologue. Rosconi *et al*. demonstrated a surprising degree of plasticity within the pan ‘essentialome’, with mechanisms for bypassing essentiality including (1) functional redundancy, (2) avoidance of toxic intermediate accumulation, (3) improved metabolite recycling and (4) pathway rewiring. Moreover, they identified that bypassing essentiality often comes at a fitness cost, an opportunity that might be exploited with strain-specific treatment strategies. For example, they found that deletion of a sugar transferase that attaches the first capsule residue onto the lipid carrier undecaprenyl phosphate suppresses the essentiality of genes further downstream in the capsule biosynthesis pathway; however, the loss of capsule renders the cell more sensitive to other stresses.

Lastly, Tn-seq can be applied to understand gene networks from a regulatory perspective [50, 51]. Fowler and Galán developed ‘FAST-INSeq’ and Smith *et al*. developed ‘SorTn-seq’, both of which involve construction of a transposon library in a strain carrying a reporter, to identify transposon insertion mutations that affect reporter expression activity on a whole-cell scale. FAST-INSeq used a chromosomally encoded reporter, replacing the coding sequence of the typhoid toxin in *S. enterica* serovar Typhi with GFP, whereas SorTn-seq used a plasmid-based reporter construct consisting of a CRISPR–Cas promoter upstream of mCherry. Both methods used FACS to sort populations of cells with altered fluorescence and identified regulatory networks underpinning the phenotype of interest. For example, Fowler and Galán applied FAST-INSeq to identify genes that altered typhoid toxin expression inside infected Henle cells and identified the PhoPQ two-component system, whereas Smith *et al*. identified that activation of the Rcs pathway (a phosphorelay system that senses envelope stress) represses CRISPR–Cas activity. Applied in this way, Tn-seq can be used to map cellular circuits and uncover the regulatory control of complex phenotypes.

## Future applications and considerations

### Transposon adaptation

Saturating transposon mutagenesis allows the insertion of functional tags almost anywhere in the genome. More recently, the Tn-seq method has been adapted to use the transposon as a translation reporter to identify translated coding sequences on a whole-genome scale independently of gene annotation [52–54]. The level of resolution gained from a Tn-seq library revealed previously undetected protein-coding sequences encoded within intergenic regions, and in some cases, these revealed operons of conserved small genes. Notwithstanding known limitations such as the limited scope to disrupt essential genes, these approaches show promise due to the scale of genome scrutiny that can be applied. Chromosomal tags are not limited to lethal selection translation reporters. It might be interesting to revisit the TnPhoA screen on a saturating scale [55], a transposon-mediated screen for determining protein topology, as the PhoA alkaline phosphatase is only functional in the periplasm, or to develop non-lethal reporters such as fluorescent proteins for *in situ* expression profiling. We can also envisage further transposon reporter adaptations for functional screening. In our earlier paper describing a saturated transposon mutant library in *E. coli* K-12 [56], we observed the phenomenon of transposon insertion to demarcate structural boundaries within proteins: a single insertion within *grpE* suggested translational read-out from the transposon and GrpE split-protein functionality. It therefore stands to reason that it would be possible to leverage saturating mutagenesis coupled with split-protein functionality to apply a bacterial two-hybrid (BACTH) screen on a whole-genome scale. BACTH uses the catalytic domain of the *Bordetella pertussis* adenylate cyclase split into two complementary fragments, T25 and T18, each fused to a bait and a prey protein of interest, respectively; if the bait and prey protein interact (or are in physical proximity *in vivo*), the reconstituted adenylate cyclase becomes functional and acts as a reporter for protein–protein interaction of the bait–prey pair [57]. We can envisage a scenario where a plasmid-encoded bait protein is coupled with a transposon-encoded split tag, to label all coding sequences *en masse* to identify proteins that interact with the bait. If plasmid-transposon bait–prey coupling can be achieved, could this approach be applied to split fluorophores or Förster resonance energy tranfer (FRET)-like assays (which measure energy transfer between fluorophores when they come into close proximity), or for monitoring drug–protein interactions using a labelled compound with a whole-protein tagging system?

### Multi-dimensional screening

We are in the infancy of dual mutagenesis screens with several dual loss-of-function (LOF) mutagenesis screens recently published [42–45], demonstrating that studies of this scale are feasible. In the future, we might expect to see the coupling of LOF with GOF mutagenesis into a single high-throughput screen, with GOF defined as a gain in functional activity either from increased expression or gene copy number. For example, the coupling of a LOF CRISPRi library with a transposon carrying an inducible outward-facing promoter (GOF) could identify overexpression mutants that can bypass essential gene knock-down. Secondly, SorTn-seq and FAST-INSeq demonstrated coupling a single reporter with genome-wide saturation mutagenesis. A combinatorial approach might incorporate a transposon-based reporter into a dual mutagenesis screen to identify gene-interaction networks for multiple reporter fusions in a genome-wide ‘SorTn-seq’ experiment. However, development of a more robust dynamic detection threshold would be needed to mitigate for genome-wide positional effects on reporter activity, which might present some challenges for data analysis and interpretation of a screen at this scale. If quantitative genome-wide reporter transposon screens can be achieved, these will be useful, for example in chemicogenomic screens, whereby global gene expression changes in response to an antibiotic (or other chemical) can be tracked *in vivo*.

Finally, higher-dimensional screens have been demonstrated with CRISPRi, using guides expressed in an array to target more than two genes in parallel [58]. One of the biggest challenges to higher-dimensional library screening is the scale of the data and the complexity of the data to be analysed. Currently, a triple or higher transposon library at saturation scale might be unrealistic given that the ‘all-to-all’ genetic interaction mapping of dual Tn-seq double mutants in *Streptococcus pneumoniae* involved ~1.4 billion mutants and sequencing of 10 billion fused barcodes [42]. However, it would be reasonable to investigate gene–gene interactions of ≥3 genes for defined targets, especially with the introduction of targeted Tn-seq (described below).

### Final remarks

One limitation of transposon screens is the requirement for many thousands of mutants when relying on random chance to achieve whole-genome coverage, in contrast with targeted mutagenesis strategies, such as CRISPR methods, that can achieve whole-genome coverage with much smaller library pools. However, ‘random’ mutagenesis and library size need not be a limiting factor for transposon screens. Arguably, the transposons used in most transposon mutagenesis screens are no longer ‘true’ transposons, but (generally) simple selection markers that make use of transposon architecture, such as inverted repeats, coupled with an exogenously provided transposase to mediate transposition. Transposase-mediated mutagenesis is just one delivery mechanism for introduction of linear fragments of DNA. With methods such as ORBIT (Oligo Recombineering followed by Bxb-1 Integrase Targeting) [59, 60], it is now possible to design entire insertional libraries, negating the need for large random library pools for tagged mutagenesis [61]. CRISPR–Cas has also been identified as a transposon delivery system [62–64] and shows promise as a functional genomics tool [65]. ‘CRISPRt’, a CRISPR-associated transposon system to programmatically disrupt target genes, has been demonstrated for targeted Tn-seq in *Acinetobacter baumannii* [66].

Tn-seq libraries have now been constructed in >100 species of bacterial species across 18 different classes and 32 orders (Table S1, available in the online Supplementary Material). The most prevalent class is Gammaproteobacteria, largely represented by *E. coli* and *Pseudomonas aeruginosa* species, with other frequently represented species including *Staphylococcus aureus*, *S. pneumoniae* and *A. baumannii*, highlighting biases from infectious disease research. However, with an ever-expanding toolkit available for genetic manipulation, Tn-seq accessibility is continually increasing. Price *et al*. demonstrated its wide utility constructing libraries in 32 diverse species, including the cyanobacterium *Synechococcus elongatus* [67]. Nevertheless, some challenges remain. Some of the barriers for library construction include extreme growth requirements, such as high temperature, pH, salinity or strict anaerobiosis, while some organisms are particularly fastidious in their growth requirements. Library construction in these species can in part be mitigated by extraction of genomic DNA and *in vitro* transposon mutagenesis, followed by harnessing natural competency to introduce the mutated genetic material back into the host. Examples of libraries constructed in this way include the notoriously recalcitrant *Neisseria gonorrhoeae* [68], the obligate anaerobe *Parvimonas micra* [69], and epibiont *Southlakia epibionticum* [70], highlighting some of the microbial diversity that can be explored with Tn-seq. Mutagenesis of species from the human microbiome have garnered interest in recent years, although transposon libraries are underrepresented within the *Firmicutes* and *Verrucomicrobia* phyla (reviewed here: [71]). One notably absent species from Tn-seq studies is the widely studied *Helicobacter pylori* [72], potentially due to its challenging culture requirements, although a small-scale (pre-2009) transposon library has been reported [73]. Other limitations to library construction might include strict barriers to horizontal gene transfer or limited competence. Finally, the presence of multiple genome copies in polyploid prokaryotes can also present additional challenges during mutant selection, as successfully integrated transposons might be recombined out of the genome if a fitness cost is imparted. Of note, Gómez-Campo *et al*. successfully constructed a transposon library in the polyploid *Thermus thermophilus* [74] but did not observe the typical insertion profile associated with essential genes.

Finally, provided a library can be constructed, arguably one of the main limitations is the interpretation of increasingly complex data. Often some prior knowledge of the phenotype is needed or integration of data with complementary ‘omics datasets’. Tn-seq is not a magic bullet method and certainly has its limitations, but the ability to disrupt almost every locus is a very powerful tool.

## Supplementary material

10.1099/mgen.0.001796 Supplementary Material 1.

## Abbreviations

BACTH: bacterial two hybrid
CRISPR: clustered regularly interspaced short palindromic repeats
CRISPRi: CRISPR interference
FACS: fluorescence-activated cell sorting
FRET: Förster resonance energy transfer
GOF: gain of function
LOF: loss of function
ORBIT: Oligo Recombineering followed by Bxb-1 Integrase Targeting
Tn-seq: transposon-insertion sequencing
